# Vietnam’s Evolving Healthcare System: Notable Successes and Significant Challenges

**DOI:** 10.7759/cureus.40414

**Published:** 2023-06-14

**Authors:** Nguyen K Quan, Andrew W Taylor-Robinson

**Affiliations:** 1 Epidemiology and Public Health, College of Health Sciences, VinUniversity, Hanoi, VNM; 2 Epidemiology and Public Health, Center for Global Health, University of Pennsylvania Perelman School of Medicine, Philadelphia, USA

**Keywords:** healthcare work force, disease prevention and control, urban and rural communities, health wellness and promotion, digital health technology, telemedicine (tm), healthcare disparity, universal health coverage (uhc), vietnam, global healthcare systems

## Abstract

From regional and rural grassroots to a nationwide level, Vietnam has established a four-tiered hierarchical healthcare system, comprising national, provincial, district, and commune healthcare centers. Over the last three decades, alongside increasing provision of universal health insurance coverage and cutting healthcare expenditure, the country has demonstrated its dedication to preventative medicine and health promotion. Recent investment in research, development, and production has led to “homegrown” vaccines for SARS-CoV-2 now undergoing clinical trial. Nevertheless, despite substantial progress in improving health outcomes for the entire population, the healthcare sector experiences significant challenges. The current public system is paper-based, requires digitalization, and lacks information technology support. In common with many other countries, there is a vast disparity in the distribution of healthcare professionals between cities and rural areas, as well as between private and public sectors. Consequently, public healthcare in remote locations is particularly underserved. Moreover, ongoing underfunding caused by high out-of-pocket expenses for the average salary, as well as stigmatization of sensitive health issues by a largely conservative populace, demand a well-articulated and culturally sensitive approach. As the level of smartphone ownership and internet coverage are both comparatively high for Southeast Asia, the introduction of telemedicine, mobile health applications, and other digital health solutions may be both practicable and beneficial. Importantly, in order to develop healthcare facilities and reduce patient direct payments, the key issue of funding must be addressed. In order to overcome disease-related stigma, a locally tailored program of community education, awareness, and engagement is required. In summary, in several ways, Vietnam provides a role model for developing healthcare systems in low- and middle-income countries. There are undoubted hurdles to overcome, but the country continues to construct a healthcare system that is accessible and affordable for the majority.

## Editorial

Introduction

Vietnam has a rapidly developing socialist-oriented market economy, commensurately increasing urbanization, and a population that, in 2023, for the first time now exceeds 100 million people. This development success story was founded on extensive political, economic, and educational reforms during the 1980s that have propelled this Southeast Asian country in a single generation from one of the world’s poorest nations to a lower middle-income economy. The gross domestic product of this unitary state is now predicted to rival developed nations by 2050 [1]. The new set of policies brought in by the Communist Party of Vietnam government was termed “Doi Moi,” a Vietnamese phrase meaning “innovation” [1]. This sought to transition from the previously ineffective command economy to “capitalized” economics. Over the years that have followed, the agriculture sector has supported economic growth and ensured food security, while access to infrastructure services, such as electricity and clean water, has risen appreciably [1]. Nevertheless, major obstacles lie ahead if the country is to maintain its rising standard of living in the face of a rapidly ageing population. Among the most fundamental issues are those relating to the healthcare sector.

There are four administrative levels of government in Vietnam: national, provincial, district, and commune. The country is divided into 63 provinces, more than 700 districts, and more than 11,000 communes [1,2]. Typically, a provincial department of health oversees between 10 and 20 districts, each of which contains dozens of communes [1,3]. The Ministry of Health manages three levels of health service delivery: primary level in districts and communes, secondary level in provinces, and tertiary level in national institutions under central government control. Working within this hierarchical system, Vietnam aims to build a solid healthcare infrastructure from the grassroots. District health centers and commune health stations are referred to as "grassroots health care systems" (primary health service delivery) [2]. Despite the fact that significant strides have been made since the Doi Moi reforms to improve health outcomes, the healthcare system faces mounting challenges to keep apace of the country’s escalating population.

Here, we highlight key strengths and notable weaknesses as perceived from an academic medical perspective. We work at Vietnam’s newest medical school, part of VinUniversity, which after only two years in operation was the youngest university in the Asia-Pacific region to receive 5-star QS ratings in seven categories. VinUniversity is owned by the country’s largest conglomerate, VinGroup. A sister subsidiary organization in this ecosystem is the Joint Commission International-accredited VinMec private healthcare system, in which clinical training is primarily based. Furthermore, with VinGroup entering into a formal alliance with the University of Pennsylvania in 2018 [4], the MD curriculum is benchmarked by and delivered in partnership with the world-renowned Perelman School of Medicine. It provides affiliate faculty who support the teaching, mostly in English, of specialist clinical curricula within the undergraduate and graduate medical education programs. As part of the international team rising to the challenge of developing the next generation of healthcare leaders in Vietnam, we share a privileged insight into where this country’s healthcare system falls short and where it excels.

Successes so far

Universal Health Coverage

Universal health coverage (UHC) is a complex and multi-faceted healthcare system, among others, including the participation of a legal framework, stakeholder engagement, health workforce, financing, and service delivery. The Vietnamese government has a strong commitment to UHC and has set a clear vision for making healthcare universal and affordable for all its citizens [5]. Vietnam has made significant progress toward achieving UHC through enacting the Law of Social Health Insurance in 2008 and its revision in 2014 [1,5]. Additionally, the government has attempted to broaden coverage for traditionally marginalized groups, such as single mothers, members of low-income households, and persons from ethnic and linguistic minorities (the Viet [Kinh] people account for 85.3% of the country's population). The latter communities are further disadvantaged by living mostly in mountainous and rural regions [3]. These efforts yielded a steady rise in health insurance coverage. As of 2021, 89.3% of Vietnamese people had personal health insurance, up from 62% in 2010. This already high proportion is expected to increase beyond 95% by 2025 [6]. Already, the Vietnam health insurance system is considered one of the most successful in Asia [7].

Affordable and Accessible Healthcare Services

Healthcare costs in Vietnam are significantly cheaper than in many other countries, making it more accessible to the general population [8]. Many community-based services, such as immunizations, hygiene, nutrition, mothers' care, and childcare, are provided free of charge or at a very low cost [9]. This aims to ensure that everyone, regardless of income, has equitable access to primary healthcare services. In addition to providing essential patient-centered healthcare, the government places a strong emphasis on health promotion and disease prevention. This includes campaigns designed to raise public knowledge and awareness of crucial health and well-being issues, such as cessation of tobacco smoking, HIV/AIDS prevention through pre- and post-exposure prophylaxis, and safe sexual practices [5]. There has been significant expenditure on public and private sector healthcare infrastructure by the government and private investors, respectively, to increase accessibility, including building new hospitals and clinics and expanding healthcare coverage to rural and remote areas [7]. As a result, healthcare services are widely available across the country, with most people living within a reasonable travelling time from a primary healthcare facility.

Health service delivery reforms initiated in Vietnam in the 1990s permitted hospitals a degree of autonomy to charge fees [10], thereby sowing the seeds for the inception of the private sector. Fast forward a quarter of a century to today, the rising popularity of private healthcare reflects the increasing demand for high-quality medical services. In advancing personalized care of a type that is well established in developed nations, the country’s private healthcare providers now offer extended consultation times, efficient diagnostic services, prompt surgical procedures using cutting-edge medical equipment, and an increasing range of mental health coverage. Unsurprisingly, accessibility and affordability are not equally distributed [8]. People on lower incomes and/or who reside in rural areas are particularly underrepresented, while the majority of private patients come from the expanding proportion of white-collar professionals who seek - and can afford - private health insurance. Therefore, the emergence of this option should be viewed in a positive light as a driver to raise national standards across the sector and as a way to reduce the burden on public hospitals. Yet, it is recognized that the societal impact will be much greater once advances in healthcare delivery and management made in the private system flow down more quickly to benefit the public system.

Emphasis on Preventive Healthcare

Preventive, community-focused healthcare not only benefits the health of the individual but also has a positive effect on the well-being of all members of the society to which they contribute [5]. Thanks to the prioritization on, and investment in, preventive medicine, Vietnam has been lauded globally for its effective response to recent pandemics such as SARS, avian influenza, and COVID-19 [11], as well as for progressively reducing the burden of infectious diseases (e.g., tuberculosis, malaria, HIV/AIDS) [12]. An allocation up to 30% of the total state budget is currently committed to the public health track [6]. Preventive healthcare is emphasized in Vietnam as a means of reducing preventable diseases (both communicable and non-communicable), enhancing health outcomes, and increasing cost-effectiveness. Policymakers are urged to prioritize preventive healthcare in the national health agenda by promoting healthy behaviors, vaccinations, regular health examinations, and early disease detection and intervention. These are performed as community outreach activities, including immunization programs, hygiene workshops, nutrition advice, and care groups for new mothers and their infants. Instigation of this framework has facilitated increased availability and enhanced standards of primary healthcare services throughout the country, as well as notably encouraging community engagement, particularly in remote and rural areas [5,7].

Medical Products, Vaccines, and Technologies

The advent of the COVID-19 pandemic has expedited major advancements in biotechnology and vaccinology, as well as truncating the timeline to global licensure of drugs and vaccines. Vietnam's healthcare system has made significant progress in terms of medical product development, vaccines, and technologies. A major advantage is the active participation and heavy investment of the private sector in establishing private hospitals and developing the pharmaceutical industry. Moreover, the rise in domestic research and development capabilities has led to notable strides forward in the manufacturing of generic drugs, vaccines, bioproducts, and other medical products [1]. Vietnam has made remarkable progress in vaccine research and production, particularly in response to COVID-19. The country has successfully developed its own vaccines (NanoCovax and CoviVac), which have undergone clinical trials (ClinicalTrials.gov Identifier NCT04922788 and NCT04830800, respectively). Vietnam is now home to more than 100 million residents, making it the world’s fifteenth most populous nation [3]. This presents logistical challenges to provide improved access to essential medicines with reduced healthcare costs, yet its pharmaceutical industry is rapidly evolving capacity to meet the growing demand. The country is inevitably prone to future public health emergencies, as there will likely be a disparity between supply and demand in drugs, vaccines, bioproducts (e.g., test kits or probes), and other medical supplies. Establishing on-site accredited biotechnology and pharmaceutical facilities will not only accelerate domestic research and development but also contribute to resolving a proportion of these logistical hurdles while optimizing cost-effectiveness [9].

Ongoing challenges

Lack of Digital Transformation of Recordkeeping

Like any other country, the healthcare system in Vietnam faces its own set of challenges. Some of these are generic issues that will resonate elsewhere, while others are uniquely shaped by the local social and cultural context. The inadequacy of technological advancements is a significant barrier. The healthcare system continues to rely primarily on manual, paper-based reporting systems [7,9]. Digitalization refers to leveraging digital technologies and digitized data (converted from manual records by the process of digitization) to enhance workflow and improve operating systems. The dearth of digitization reduces the agility and flexibility with which data are integrated, thus limiting insights into healthcare outcomes, resource allocation, and patient requirements. The delayed introduction of technological advancements and digitalization into Vietnam's healthcare system, particularly in the public sector, results in a current insufficiency of real-time data for tracking improvement, making it difficult to effectively administer the system and inform decisions [13,14]. As “what gets measured gets done,” the ambitious healthcare reform goals set by the Vietnamese government cannot be met without accurate data on progress. Hence, digital transformation is critically required to bring medical record keeping - and therefore opportunities for improved patient care, outcomes, and experiences - into the digital age.

Imbalanced Distribution of the Healthcare Workforce

In order to support the health and well-being of Vietnam’s growing population requires enlargement of the professional workforce within the healthcare sector, especially in high-density urban areas. According to the most recently available statistics, the number of medical doctors in Vietnam increased significantly over the 35 years between 1986 and 2021, from 15,000 to more than 109,500 [6]. This has been achieved through a phased expansion of medical universities to deliver accredited MD programs. In parallel, the government has introduced various postgraduate training programs for the entire spectrum of healthcare workers, including continuous professional development to improve their specialist knowledge and practical skills.

Nevertheless, the overall rise in the numbers of qualified healthcare workers does not satisfactorily address a pronounced geographical imbalance in their distribution. Relative to population density, a disproportionately high concentration of healthcare professionals work in metropolitan centers, while rural and remote areas often suffer from a shortage of healthcare professionals [6]. Of course, the reluctance of healthcare workers to relocate from cities to countryside is a phenomenon that is not unique to Vietnam, being noted in both economically developing and developed countries. In order to redress the imbalance in healthcare worker dispersal, the national and provincial governments, together with local healthcare organizations, have implemented various initiatives [7,13,14]. One strategy is to offer incentives for healthcare workers to relocate to rural and remote locations. These incentives usually include higher salaries and housing subsidies. In addition, telemedicine has developed and become a viable option for providing general practitioner consultations and other primary healthcare services to traditionally underserved areas. Telemedicine, which was initially intended as a temporary solution to address the consequences of social distancing and healthcare overburden caused by COVID-19, is now a promising alternative for several health issues. This technology enables healthcare professionals to remotely diagnose and treat patients using video conferencing, smart devices, and other digital tools [15,16]. Another approach is to train and equip local community leaders as voluntary health support workers who are approved to offer basic healthcare services in their own villages.

Underfunding and High Out-of-Pocket Payments

The Vietnam healthcare system is chronically underfunded, at least in the majority public sector. In particular, insufficient resources are allocated to healthcare infrastructure, equipment, and staff training. Annually, the total budget for health falls below the expenditure required to meet the sector’s needs. This has resulted in a shortage of medical supplies and equipment, inadequate incentives for healthcare workers, and restricted research and development. Moreover, it leads to high fees (relative to the cost of living) passed directly onto patients, which is a major challenge for many Vietnamese citizens. According to the World Health Organization, in 2016, out-of-pocket payments accounted for 41% of total health expenditure in the country [2]. This can be a significant financial burden for low-income families who are unable to afford expensive medical treatments, which may result in delayed or inadequate care. Well-off patients still prefer private hospitals and are willing to dig into their own pockets for the privilege despite the fact that the public sector is more likely to provide a better range of services and post-treatment care in the community [1,7]. In order to remedy this deficit, policymakers should prioritize healthcare funding and explore financing mechanisms that are innovative, at least for a socialist state, such as brokering public-private partnerships and attracting independent investors. A potential solution to one of the issues raised here is to externally fund the installation of digital technology in public hospitals, the application of which may be used to efficiently schedule appointments, thereby reducing waiting times and optimizing outpatients’ experiences. This is just one example of the benefit of improving the efficiency of public hospitals by investing in technology and streamlining processes. There is also a need to raise the productivity of healthcare delivery through better management practices. By tackling what is evidently a “wicked problem” (so-called because it is pervasive, complex, and intransigent), Vietnam’s healthcare system can become more resilient and better equipped to meet its present and future needs.

Social Stigmatization of Health Issues and Healthcare Workers

Vietnam is a socially conservative country where health concerns are generally shared only among close family, if at all. In this cultural context in which privacy is prized, such conditions as cancer, female health issues, sexually transmitted infections, and infectious diseases associated with poverty are all taboo subjects, discussion of which, even in a medical context, is highly sensitive. Hence, the social stigmatization of people with one of these or another health issue, and even of healthcare workers treating them, is a significant impediment to efficient healthcare services and negatively affects patient outcomes. While younger generations are more open to discussing health matters, negative stereotyping and misinformation remain toward some infectious diseases, leading to ostracization or cyberbullying of, for instance, persons with HIV, tuberculosis, or chlamydia [17-19]. HIV/AIDS continues to be stigmatized, with many people harboring negative attitudes toward people living with HIV who they know are receiving antiretroviral treatment. Moreover, mental illness of any kind is almost entirely unaccepted, resulting in severe discrimination and social exclusion of those with identified or assumed mental health issues [20]. In a culture in which the predominant view is unsympathetic and considers even stress-related illness as a weakness of character, the fear of being socially stigmatized can prevent people, particularly from ethnic minorities, from seeking appropriate medical care. Inevitably, this leads to delayed diagnosis and treatment. Overall, addressing deep-rooted social stigma toward both physical and mental health issues, and which affects attendant healthcare workers, is crucial to improving the quality of healthcare services and patient outcomes.

Conclusions

Vietnam has a swiftly developing market-based economy, which so far this century has witnessed substantial improvements in food security, infrastructure services, and the public governance framework. The nation has demonstrated its adherence to a policy of preventive medicine and health promotion by enhancing UHC with insurance and decreasing the cost of healthcare services. Moreover, a recent pandemic-driven commitment to research, development, and manufacturing of biomedical products and healthcare technology is a welcome advance. Even so, a 2023 “health of the nation” report card reveals much room for improvement. There is a lack of digitalization and technological integration in the healthcare system, which continues to rely heavily on paper-based and manual reports, especially in the majority public sector. Significant disparities exist in staffing levels and quality of care between urban and rural settings, as well as between private and public sectors.

## References

[1] Le DC, Kubo T, Fujino Y, Pham TM, Matsuda S (2010). Health care system in Vietnam: current situation and challenges. Asian Pacific J Dis Manag.

[2] (2023). World Health Organization: Western Pacific country profile, Viet Nam. Healthcare system administration. https://www.who.int/vietnam/vi/health-topics/health-systems-governance.

[3] (2023). Vietnam General Statistics Office: Completed Results of the 2019 Viet Nam Population and Housing Census. https://www.gso.gov.vn/wp-content/uploads/2019/12/Ket-qua-toan-bo-Tong-dieu-tra-dan-so-va-nha-o-2019.pdf.

[4] (2023). University of Pennsylvania, Perelman School of Medicine. VinGroup-Penn Alliance. https://www.med.upenn.edu/vingroup-penn-alliance/.

[5] Nguyen TK, Cheng TM (2014). Vietnam's health care system emphasizes prevention and pursues universal coverage. Health Aff (Millwood).

[6] (2023). Viet Nam General Statistics Office: Statistical Yearbook of Viet Nam. Nam.

[7] Somanathan A: What lies ahead in (2023). What lies ahead in Vietnam’s path towards Universal Health Coverage? East Asia & Pacific on the Rise. https://blogs.worldbank.org/eastasiapacific/what-lies-ahead-vietnam-path-towards-universal-health-coverage.

[8] Nguyen KT, Khuat OT, Ma S, Pham DC, Khuat GT, Ruger JP (2012). Impact of health insurance on health care treatment and cost in Vietnam: a health capability approach to financial protection. Am J Public Health.

[9] Quan NK, Anh NL, Taylor-Robinson AW (2023). The global COVID-19 vaccine surplus: tackling expiring stockpiles. Infect Dis Poverty.

[10] (2023). World Health Organization: Western Pacific country profile, Viet Nam. Hospitals in Viet Nam. https://www.who.int/vietnam/health-topics/hospitals.

[11] Tran TP, Le TH, Nguyen TN, Hoang VM (2020). Rapid response to the COVID-19 pandemic: Vietnam government's experience and preliminary success. J Glob Health.

[12] Kato M, Long NH, Duong BD (2014). Enhancing the benefits of antiretroviral therapy in Vietnam: towards ending AIDS. Curr HIV/AIDS Rep.

[13] Kien VD, Van Minh H, Giang KB, Ng N, Nguyen V, Tuan LT, Eriksson M (2018). Views by health professionals on the responsiveness of commune health stations regarding non-communicable diseases in urban Hanoi, Vietnam: a qualitative study. BMC Health Serv Res.

[14] (2023). The World Bank: A Review of Science, Technology and Innovation in Vietnam. https://www.worldbank.org/en/country/vietnam/publication/a-review-of-science-technology-and-innovation-in-vietnam.

[15] Dat TV, Tu VL, Quan NK (2023). Telepharmacy: a systematic review of field application, benefits, limitations, and applicability during the COVID-19 pandemic. Telemed J E Health.

[16] Caffery LA, Muurlink OT, Taylor-Robinson AW (2022). Survival of rural telehealth services post-pandemic in Australia: a call to retain the gains in the 'new normal'. Aust J Rural Health.

[17] Pham HN, Protsiv M, Larsson M, Ho HT, de Vries DH, Thorson A (2012). Stigma, an important source of dissatisfaction of health workers in HIV response in Vietnam: a qualitative study. BMC Health Serv Res.

[18] Redwood L, Fox GJ, Nguyen TA (2022). Good citizens, perfect patients, and family reputation: Stigma and prolonged isolation in people with drug-resistant tuberculosis in Vietnam. PLOS Glob Public Health.

[19] Nguyen SH, Dang AK, Vu GT (2019). Lack of knowledge about sexually transmitted diseases (STDs): implications for STDs prevention and care among dermatology patients in an urban city in Vietnam. Int J Environ Res Public Health.

[20] Ta TM, Zieger A, Schomerus G (2016). Influence of urbanity on perception of mental illness stigma: a population based study in urban and rural Hanoi, Vietnam. Int J Soc Psychiatry.

